# Opening of the Medical Colleges

**Published:** 1872-10-01

**Authors:** 


MEDICAL EXAMINER.

*A Semi-Monthly Journal of Medical Sciences.*

EDITED BY

N. S. DAVIS, M. D., AND F. H. DAVIS, M. D.

Chicago, October 1st, 1872.

### EDITORIAL.

### OPENING OF THE MEDICAL COLLEGES.

THE CHICAGO MEDICAL COLLEGE opened its regular annual course of instruction for 1872–3. on Tuesday evening, October 1st, in the amphitheatre of the College. The room was well filled with an audience of students and citizens.

The introductory lecture was given by Prof. D. T. Nelson, who made use of the occasion to give a very interesting review of the subject of bioplasm, or the nature and origin of germinal matter. The term opened with a decided increase in the number of students over previous years, and with important additional facilities for instruction, both clinical and didactic.

RUSH MEDICAL COLLEGE.—This institution commenced its annual session on Wednesday evening, October 2d, in the lecture room of the temporary college building on 18th street. The introductory lecture was given by Prof. J. A. Allen.

How the present class compares in numbers with former classes we have not learned.

The WOMAN'S HOSPITAL MEDICAL COLLEGE commenced its session a few days earlier than either of the others.

The opening lecture was given by Prof. Mary H. Thompson. The class of ladies in attendance is small. This College and Hospital is now permanently located in the western border of the city, at the corner of West Adams and Paulina streets, and is worthy of the patronage of such ladies as are determined to educate themselves for the profession of medicine.

IT will be seen that, though the great fire of October 9, 1871, has resulted in changing the location of some of our colleges, it has neither extinguished nor seriously crippled any of them; and Chicago to-day, with its numerous and well manned hospitals and dispensaries, affords as complete and well arranged facilities for medical students to acquire a thorough knowledge of every department of medical science and art, as any city in our country.

